# Evaluation of the United Kingdom‐primary biliary cholangitis and global primary biliary cholangitis group prognostic models for primary biliary cholangitis patients treated with ursodeoxycholic acid in the U.S. population

**DOI:** 10.1002/jgh3.12223

**Published:** 2019-07-22

**Authors:** Mohammad Alomari, Fahrettin Covut, Laith Al Momani, Pravallika Chadalavada, Asif Hitawala, Mark F Young, Carlos Romero‐Marrero

**Affiliations:** ^1^ Department of Internal Medicine Cleveland Clinic Foundation Cleveland Ohio USA; ^2^ Department of Internal Medicine East Tennessee State University Johnson City Tennessee USA; ^3^ Department of Gastroenterology and Hepatology East Tennessee State University Johnson City Tennessee USA; ^4^ Department of Gastroenterology Hepatology and Nutrition, Cleveland Clinic Foundation Cleveland Ohio USA

**Keywords:** biliary, cholangitis, cirrhosis, overlap syndrome, prognosis, ursodeoxycholic acid

## Abstract

**Background and Aim:**

The United Kingdom‐primary biliary cholangitis (UK‐PBC) and global primary biliary cholangitis group (GLOBE) prognostic models have been recently developed to predict long‐term outcomes in primary biliary cholangitis (PBC). However, these predictive scores have not yet been well evaluated in the U.S. population.

**Methods:**

We retrospectively reviewed newly diagnosed PBC patients at the Cleveland Clinic between November 1998 and February 2017. Adverse events were defined as liver transplantation, liver‐related mortality, and all‐cause mortality. Transplant‐free survival (TFS) was estimated using the Kaplan–Meier method. Predictive performances of all prognostic models were evaluated using the C‐statistic.

**Results:**

We identified 352 patients who used ursodeoxycholic acid therapy. Of them, 311 (88.4%) only had PBC, while 41 (11.6%) were diagnosed with PBC‐autoimmune hepatitis overlap. A total of 22 (6%), 47 (13%), and 55 (16%) patients had adverse events within 5, 10, and 15 years after diagnosis, respectively. In patients with PBC only, the C‐statistic in predicting 15‐year adverse events was 0.75 per GLOBE compared to 0.74 per UK‐PBC (*P* = 0.94), 0.73 per Rotterdam (*P* = 0.44), 0.66 per Barcelona (*P* = 0.004), 0.65 per Paris 1 (*P* = 0.005), 0.62 per Paris 2 (*P* < 0.0001), 0.60 per Toronto (*P* < 0.0001), and 0.60 per Mayo (*P* < 0.0001) scores. Median follow‐up was 9.2 years. Ten‐year TFS for patients who had optimal *versus* suboptimal treatment response was 92 *versus* 74% per Paris 1 (*P* < 0.0001), 95 *versus* 79% per Paris 2 (*P* = 0.0002), 93 *versus* 65% per Barcelona (*P* < 0.0001), and 96 *versus* 68% per Rotterdam (*P* < 0.0001) risk scores, respectively.

**Conclusion:**

In our cohort of PBC patients, the UK‐PBC and GLOBE scores were both accurate and reasonably valid prognostic models in the U.S. population.

## Graphical abstract

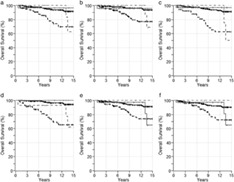

In this study, we evaluated the performance of newly developed prognostic models, the United Kingdom‐primary biliary cholangitis and global primary biliary cholangitis group scores, to predict long‐term outcomes in patients with primary biliary cholangitis treated with ursodeoxycholic acid, adding to the expanding corpus of literature that discusses the reliability and accuracy of using those models outside Europe. Our findings suggest that advanced prognostic models are reliable and reasonably accurate to predict adverse events and can thus be generalized to the U.S. population.

## Introduction

Primary biliary cholangitis (PBC), formerly known as primary biliary cirrhosis,^1^ is an autoimmune liver disease characterized by progressive lymphatic destruction of small intrahepatic biliary ducts causing cholestasis. In the absence of effective therapy, it frequently progresses to cirrhosis, which usually poses a poor prognosis with a high risk of liver‐related complications and death.^2^ Previous studies have reported a wide‐ranging prevalence of PBC from 19 to 402 cases per million population,^3^ with a higher incidence in women (female to male ratio 8:1), typically presenting between 30 and 65 years of age.^4^, ^5^

The etiology of PBC is postulated to be due to an interplay of genetic predisposition and environmental triggers.^6^ Ursodeoxycholic acid (UDCA) therapy has been the first‐line standard of care for PBC patients since the Food And Drug Administration (FDA) approval in 1997 with a recommended dose of 13–15 mg/kg. It acts by modifying the bile acid pool and reducing the levels of pro‐inflammatory cytokines and vasoactive mediators, thereby preventing apoptosis and cellular necrosis.^7^ UDCA has been proven to be well‐tolerated and highly effective in delaying progression to end‐stage liver disease (ESLD), prolonging transplant‐free survival (TFS) and overall survival (OS).^8^, ^9^, ^10^ An alternative or adjunctive therapy with obeticholic acid, a farnesoid X receptor antagonist, has been approved by the FDA in May 2016 for UDCA nonresponders.^11^, ^12^ Other medications such as colchicine, methotrexate, and mycophenolate mofetil are currently under investigation.^13^, ^14^

Multiple prognostic scores have been proposed by various medical centers, mainly from Europe, to identify biochemical and clinical predictors of survival after treatment with UDCA, such as the Mayo risk score,^15^ Rotterdam criteria,^16^ Paris 1 criteria,^17^ Paris 2 criteria,^18^ Toronto criteria,^19^ and Barcelona criteria.^20^ However, all of these scoring systems were developed and validated in a local population, thus resulting in varied outcomes and limitations due to the complexity of PBC.^21^

Recently, two advanced scoring systems, the United Kingdom‐primary biliary cirrhosis (UK‐PBC) and the global primary biliary cholangitis group (GLOBE) scores, have been developed using biochemical parameters at baseline and 1 year after the introduction of UDCA therapy. The UK‐PBC score was developed in 2016 from a multicenter cohort of 1916 patients and validated in a multicenter cohort of 1249 patients in the United Kingdom.^22^ In contrast, the GLOBE score was developed by combining raw data from the above medical centers in addition to several other countries, including Italy, Japan, and the United States. The GLOBE score demonstrated a better C‐statistic in derivation and validation cohorts compared to the other scores. The GLOBE score predicted 5‐year and 10‐year survival, with positive predictive values (PPVs) of 98 and 88%, respectively.^23^ However, the reference population data were derived only from the Netherlands, which might affect the external validity of this scoring system. Notably, the GLOBE and UK‐PBC scores provide more precise and personalized estimates of the risk of developing ESLD within defined time points in contrast to the other existing long‐term prognostic models that dichotomize patients into being at low risk (treatment responders) or high risk (treatment nonresponders) of developing ESLD at any point in time.

Use of the aforementioned advanced prognostic models in patients with PBC has become a standard method to manage, monitor, and risk stratify UDCA users.^24^ The advancements of liver transplant and the development of alternative effective therapies for UDCA nonresponders have led to the wider use of those biomarkers.^25^ For example, the application of those prognostic biomarkers allows for appropriate candidate selection of second‐line therapies and aids in identifying high‐risk patients for a closer follow‐up or the consideration of liver transplant.

The current literature lacks any studies that externally validate these scoring systems exclusively in the American population. Therefore, we conducted a retrospective study at our tertiary center with complete biochemical data in an attempt to externally validate the UK‐PBC and GLOBE scoring systems for prognostication of PBC among UDCA users in the U.S. population.

## Methods

### *Patient selection and treatment protocols*

Electronic medical records of newly diagnosed PBC patients between 1998 and 2017 at the Cleveland Clinic were reviewed. All patients were treated with 13–15 mg/kg daily of UDCA. We excluded patients (i) younger than 18 years of age (*n* = 53); (ii) those with concomitant alternative causes of liver disease other than autoimmune hepatitis (*n* = 111); (iii) those with documented risky alcohol use within a year before PBC diagnosis (*n* = 82); (iv) those with unavailable liver function tests at baseline or 1 year after initiation of UDCA (*n* = 182); and (v) those unable to complete at least 1 year of UDCA due to side effects, loss of follow‐up, need of liver transplantation, or death (*n* = 265). The protocol was approved by the institutional review board of the Cleveland Clinic.

### *Diagnostic criteria, surveillance, and definitions*

PBC was diagnosed in the presence of at least two of the following three criteria^1^: serum alkaline phosphatase (ALP) levels at least 1.5 times the upper limit of normal,^2^ positive test for antimitochondrial antibody (AMA), and^3^ histological manifestations of portal area inflammation and bile duct injury.^6^ Histological staging of liver biopsy was performed as previously defined.^26^

Autoimmune hepatitis–primary biliary cholangitis (AIH‐PBC) overlap syndrome was diagnosed in patients who fulfilled the PBC diagnostic criteria in addition to two or more AIH criteria^1^: alanine aminotransferase (ALT) levels more than five times the upper limit of normal,^2^ serum immunoglobulin G levels more than two times the upper limit of normal or a positive test for antismooth muscle antibodies, and^3^ liver biopsy showing moderate or severe periportal or periseptal lymphocytic piecemeal necrosis or interface hepatitis.

Patients were followed up every 3–6 months with complete blood count, basic metabolic panel, liver enzymes, albumin, and bilirubin. Those with advanced‐stage liver disease were followed for prothrombin time, international normalized ratio, and alpha‐fetoprotein as well. If indicated, hepatocellular carcinoma (HCC) surveillance was performed with liver ultrasonography every 6 months, with HCC diagnosis by multiphase imaging and/or histology. Cirrhosis was diagnosed with clinical evidence of portal hypertension, hepatic decompensation, radiographic evidence of liver nodularity, or liver biopsy.^27^

Adverse events were defined as liver transplantation or death from liver‐related causes such as liver decompensation and HCC. All‐cause mortality was considered an adverse event for the GLOBE score.^23^ Paris 1, Paris 2, Barcelona, Rotterdam, Toronto, Mayo, GLOBE, and UK‐PBC prognostic models were used to assess response to UDCA and predict adverse events.

### *Statistical analysis*

Baseline statistics were compared between patients with and without adverse events using two sample T‐tests for continuous variables such as age, weight, body mass index, and laboratory values. The Chi‐square test or Fisher's exact test was used for categorical variables such as gender, ethnicity, treatment response, and cirrhosis. OS was calculated from the date of diagnosis to the date of death from any cause, and TFS was calculated from the date of diagnosis to the date of liver transplantation or liver‐related death. Both were estimated using the Kaplan–Meier method and compared using the log‐rank test. Predictive performances of all prognostic models for adverse events were evaluated using the C‐statistic representing the area under the receiver operating characteristic (ROC) curve, with larger values indicating better discrimination. All statistical calculations were made using R statistical software version 3.4.0 (R Foundation for Statistical Computing, Vienna, Austria).

## Results

### *Patient characteristics*

**Figure 1:**
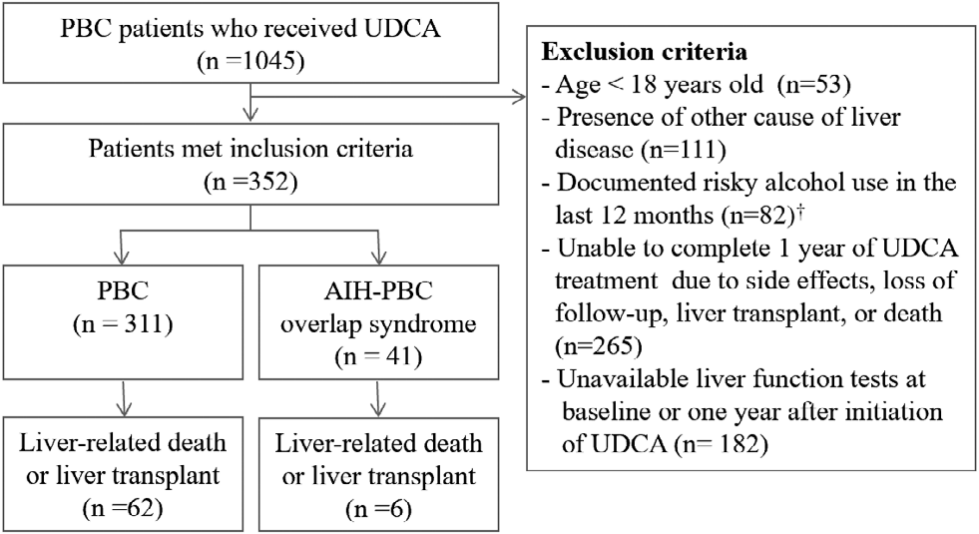
Flow diagram of patient selection and treatment outcomes. ^†^As per the National Institute on Alcohol Abuse and Alcoholism definition: Men under age 65: >14 drinks/week or >4 drinks/day. Women and adults 65 years and older >7 drinks/week or >3 drinks/day. AIH‐PBC, autoimmune hepatitis–PBC, primary biliary cholangitis; UDCA, ursodeoxycholic acid.

Between November 1998 and February 2017, 1045 patients with newly diagnosed PBC were screened, and 693 patients were excluded as illustrated in Figure 1; hence, 352 patients were included for analysis. Patient and treatment characteristics are detailed in Table 1. The median age at diagnosis was 55 years (range: 18–88); 50 (14%) patients were male, and 319 (91%) were Caucasian. Forty‐one (12%) patients were diagnosed with AIH‐PBC overlap syndrome. AMA was positive for 313 (89%) patients, and smooth muscle antibodies were positive for 65 (26%) of 252 patients who were tested for it. A total of 112 (18%) patients had cirrhosis at the time of diagnosis. Of 259 (74%) patients who had a liver biopsy at diagnosis, an actual biopsy report was available to review for 196 (56%) patients. Of these 196 patients, 7 (3%) had stage 0, 109 (56%) had stage 1 or 2 and 80 (41%) had stage 3 or 4 disease.

**Table 1:** Patient and treatment characteristics

Characteristics	All patients, *n* (%)	Patients with adverse events†, *n* (%)	Patients without adverse events†, *n* (%)	*P* value
Demographics				
Age (year), median (range)	55 (6–88)	52 (14–88)	55 (6–87)	0.64
Gender: female	302 (85.8)	54 (79.4)	248 (87.3)	<0.0001
Caucasian ethnicity	319 (91)	59 (87)	260 (92)	0.22
Body mass index (kg/m^2^), median (range)	27 (13–54)	27 (17–53)	27 (13–54)	0.35
Weight (kg), median (range)	67 (30–100)	69 (39–100)	66 (30–94)	0.014
Type of liver disease				0.42
Primary biliary cirrhosis	311 (88.4)	62 (91.2)	249 (87.7)	
Overlap syndrome	41 (11.6)	6 (8.8)	35 (12.3)	
Laboratory findings at diagnosis				
Platelet (10^9^/L), median (range)	222 (7–713)	131 (7–574)	231 (20–713)	<0.0001
Albumin (g/dL), median (range)	40 (17–54)	33 (19–45)	41 (17–54)	<0.0001
Bilirubin (mg/dL), median (range)	0.6 (0.1–32.2)	2.2 (0.2–13.3)	0.5 (0.1–32.2)	<0.0001
AST/ALT (U/L), median (range)	34 (6–694)	40 (12–694)	32 (6–596)	0.037
ALP (U/L), median (range)	156 (32–1658)	187 (47–1658)	150 (32–1520)	0.028
AMA positivity	313 (89)	60 (88)	253 (89)	0.26
SMA positivity‡	65/252 (25.8)	10/37 (27.0)	55/215 (25.6)	0.85
Treatment response				
Paris 1	258 (73.3)	34 (50.0)	224 (78.9)	<0.0001
Paris 2	195 (55.4)	25 (36.8)	170 (59.9)	0.001
Barcelona	292 (83.0)	39 (57.4)	253 (89.1)	<0.0001
Rotterdam	254 (72.2)	23 (33.8)	231 (81.3)	<0.0001
Toronto§	251 (71.3)	42 (61.8)	209 (73.6)	0.053
Mayo	269 (76.4)	45 (66.2)	224 (78.9)	0.027
Cirrhosis	112 (31.8)	50 (73.5)	62 (21.8)	<0.0001
Hepatic encephalopathy	62 (17.6)	37 (54.5)	25 (8.8)	<0.0001
Varices	78 (22.2)	37 (54.5)	41 (14.4)	<0.0001
Ascites	63 (17.9)	32 (47.1)	31 (10.9)	<0.0001

† Adverse event was defined as liver transplantation or liver‐related death at any time.

‡ Only 252 (72%) patients were tested.

§ Response according to Toronto criteria calculated after 2 years.

ALP, alkaline phosphatase; ALT, alanine aminotransferase; AMA, antimitochondrial antibody; AST, aspartate aminotransferase; SMA, smooth muscle antibody.

### *Evaluation of treatment response and validation of prognostic models*

The UDCA treatment response rate was 73% per Paris 1, 55% per Paris 2, 83% per Barcelona, 72% per Rotterdam, 71% per Toronto, and 76% per Mayo criteria. A total of 22 (6%), 47 (13%), and 55 (16%) patients had adverse events within 5, 10, and 15 years after diagnosis, respectively.

Area under the ROC curve in predicting 15‐year adverse events for patients with PBC only was 0.75 (95% confidence interval [CI]: 0.66–0.83) per GLOBE score compared to 0.74 (95% CI: 0.66–0.83, *P* = 0.94) per UK‐PBC, 0.73 (95% CI: 0.66–0.80, *P* = 0.44) per Rotterdam, 0.66 (95% CI: 0.58–0.73, *P* = 0.004) per Barcelona, 0.65 (95% CI: 0.57–0.72, *P* = 0.005) per Paris 1, 0.62 (95% CI: 0.55–0.69, *P* < 0.0001) per Paris 2, 0.60 (95% CI: 0.52–0.67, *P* < 0.0001) per Toronto, and 0.60 (95% CI: 0.52–0.67, *P* < 0.0001) per Mayo scores (Table 2).

**Table 2:** Predictive performances of prognostic models for adverse events in patients with primary biliary cholangitis

	Paris 1	Paris 2	Barcelona	Rotterdam	Toronto	Mayo	GLOBE	UK‐PBC
Event within 5 years								
C‐statistic	0.62	0.57	0.64	0.74	0.54	0.52	0.73	0.70
95% CI	0.50–0.73	0.45–0.68	0.53–0.75	0.64–0.84	0.43–0.65	0.42–0.62	0.61–0.86	0.57–0.82
Sensitivity (%)	48	57	43	71	38	29	—	—
Specificity (%)	76	56	85	76	70	76	—	—
PPV (%)	12	9	17	18	9	8	—	—
NPV (%)	95	95	95	97	94	94	—	—
Event within 10 years								
C‐statistic	0.66	0.63	0.67	0.75	0.59	0.59	0.75	0.75
95% CI	0.58–0.74	0.55–0.71	0.60–0.75	0.67–0.82	0.51–0.67	0.51–0.67	0.65–0.84	0.66–0.84
Sensitivity (%)	53	67	47	70	47	40	—	—
Specificity (%)	78	59	88	80	72	78	—	—
PPV (%)	28	21	38	36	21	22	—	—
NPV (%)	91	92	91	94	89	89	—	—
Event within 15 years								
C‐statistic	0.65	0.62	0.66	0.73	0.60	0.60	0.75	0.74
95% CI	0.57–0.72	0.55–0.69	0.58–0.73	0.66–0.80	0.52–0.67	0.52–0.67	0.66–0.83	0.66–0.83
Sensitivity (%)	51	65	43	65	50	41	—	—
Specificity (%)	79	59	88	80	73	78	—	—
PPV (%)	31	23	40	38	24	26	—	—
NPV (%)	90	90	89	93	88	88	—	—

CI, confidence interval; GLOBE, global primary biliary cholangitis group; NPV, negative predictive value; PPV, positive predictive value; UK‐PBC, United Kingdom‐primary biliary cholangitis.

For patients with AIH‐PBC overlap syndrome, area under the ROC curve in predicting 15‐year adverse events was 0.79 (95% CI: 0.61–0.97) per Rotterdam score compared to 0.71 (95% CI: 0.49–0.94, *P* = 0.39) per GLOBE, 0.68 (95% CI: 0.45–0.91, *P* = 0.31) per Barcelona, 0.66 (95% CI: 0.35–0.97, *P* = 0.36) per UK‐PBC, 0.61 (95% CI: 0.38–0.84, *P* = 0.10) per Paris 1, 0.55 (95% CI: 0.32–0.78, *P* = 0.033) per Paris 2, and 0.51 (95% CI: 0.34–0.69, *P* = 0.014) per Mayo scores (Table 3).

**Table 3:** Predictive performances of prognostic models for adverse events in patients with overlap syndrome

	Paris 1	Paris 2	Barcelona	Rotterdam	Toronto	Mayo	GLOBE	UK‐PBC
Event within 5 years								
C‐statistic	0.34	0.29	0.40	0.84	0.41	0.43	0.65	0.88
95% CI	NA	NA	NA	NA	NA	NA	NA	NA
Sensitivity (%)	0	0	0	100	0	0	—	—
Specificity (%)	68	58	80	68	83	85	—	—
PPV (%)	0	0	0	7	0	0	—	—
NPV (%)	96	96	97	100	97	97	—	—
Event within 10 years								
C‐statistic	0.46	0.41	0.53	0.73	0.54	0.56	0.58	0.53
95% CI	0.21–0.72	0.15–0.67	0.28–0.78	0.47–0.98	0.29–0.80	0.31–0.81	0.32–0.84	0.19–0.88
Sensitivity (%)	25	25	25	75	25	25	—	—
Specificity (%)	68	57	81	70	84	86	—	—
PPV (%)	8	6	13	21	14	17	—	—
NPV (%)	89	88	91	96	91	91	—	—
Event within 15 years								
C‐statistic	0.61	0.55	0.68	0.79	0.50	0.51	0.71	0.66
95% CI	0.38–0.84	0.32–0.78	0.45–0.91	0.61–0.97	0.32–0.67	0.34–0.69	0.49–0.94	0.35–0.97
Sensitivity (%)	50	50	50	83	17	17	—	—
Specificity (%)	71	60	86	74	83	86	—	—
PPV (%)	23	18	38	36	14	17	—	—
NPV (%)	89	88	91	96	85	86	—	—

CI, confidence interval; GLOBE, global primary biliary cholangitis group; NA, not available; NPV, negative predictive value; PPV, positive predictive value; UK‐PBC, United Kingdom‐primary biliary cholangitis.

### *Overall and TFS*

**Figure 2:**
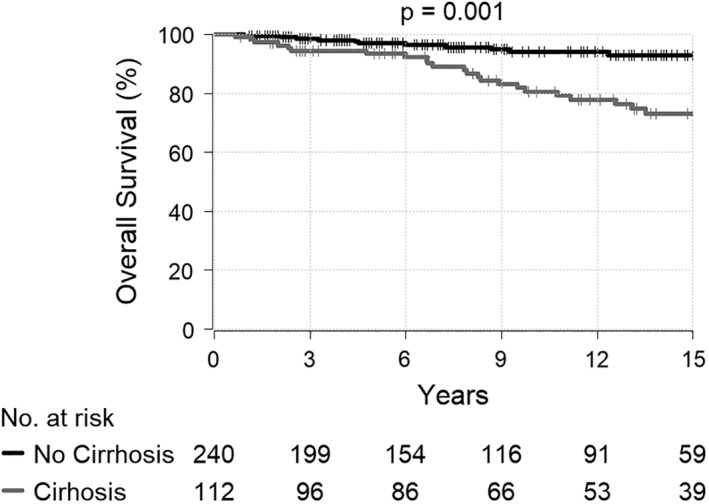
Overall survival of patients with baseline cirrhosis (gray line) and without baseline cirrhosis (black line).

Median follow‐up time was 9.2 years from diagnosis. The 10‐year OS for patients with and without baseline cirrhosis was 81% (95% CI: 72.9–89.2) and 94% (95% CI: 90.5–97.8), respectively (*P* = 0.0011) (Fig. 2).

**Figure 3:**
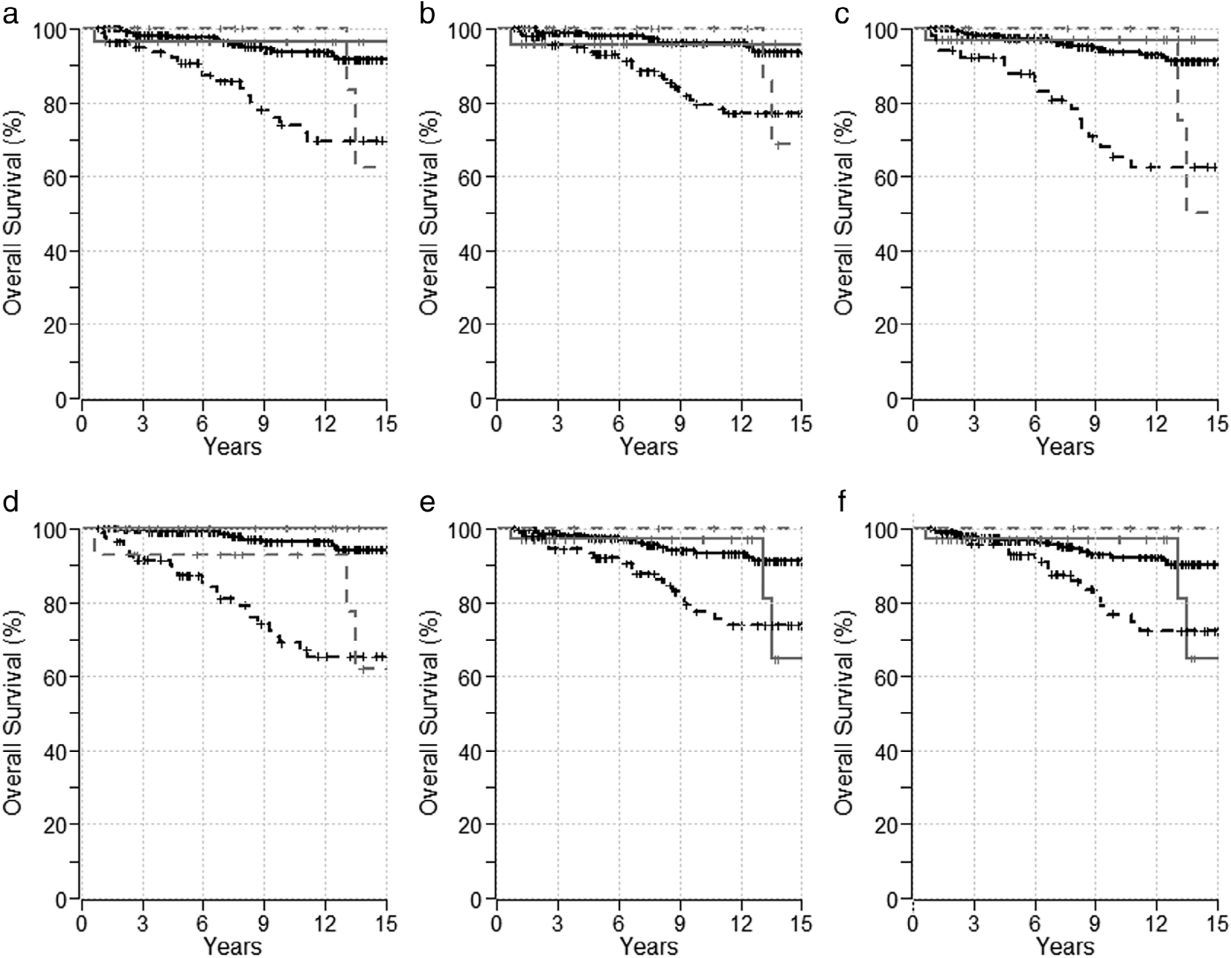
Overall survival of patients with primary biliary cholangitis (black line) and overlap syndrome (gray line) in response (‐‐‐‐‐ line) and no response (‐ ‐ ‐ line) cohorts based on risk scores. (a) Paris‐1, (b) Paris‐2, (c) Barcelona, (d) Rotterdam, (e) Toronto, and (f) Mayo.

The 10‐year OS for patients with PBC who had an optimal *versus* suboptimal treatment response was 93% (95% CI: 89.8–97.4) *versus* 74% (95% CI: 63.5–86.2) per Paris 1 (*P* < 0.0001), 96% (95% CI: 92.9–99.6) *versus* 80% (95% CI: 72.1–87.7) per Paris 2 (*P* = 0.0002), 94% (95% CI: 90.2–97.3) *versus* 65% (95% CI: 52.4–81.5) per Barcelona (*P* < 0.0001), 96% (95% CI: 93.3–99.3) *versus* 69% (95% CI: 58.6–81.2) per Rotterdam (*P* < 0.0001), 93% (95% CI: 89.2–97.2) *versus* 78% (95% CI: 68.1–88.2) per Toronto (*P* = 0.0008), and 92% (95% CI: 88.2–96.3) *versus* 77% (95% CI: 66.3–89.1) per Mayo (*P* = 0.003) risk scores (Fig. 3).

**Figure 4:**
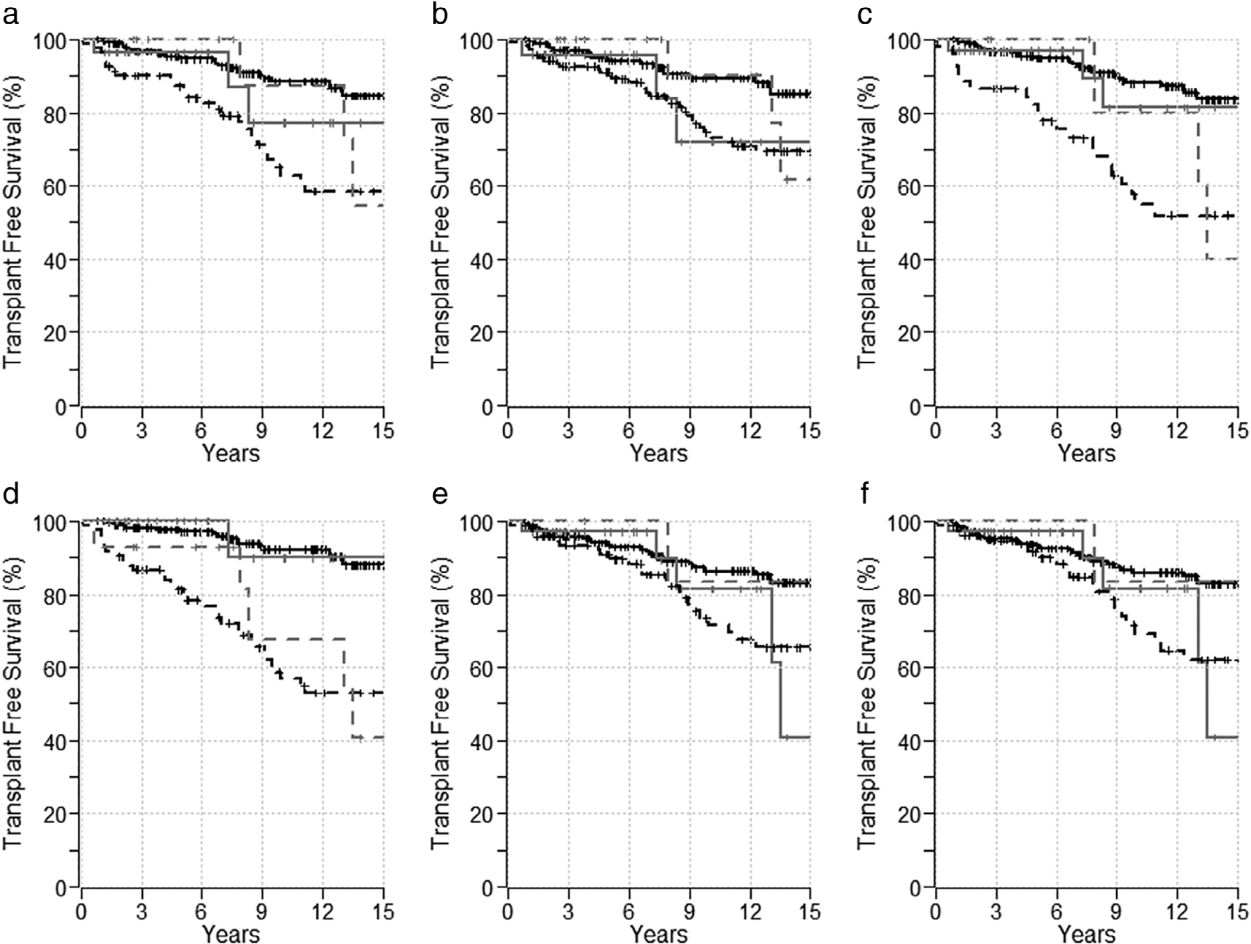
Transplant‐free survival of patients with primary biliary cholangitis (black line) and overlap syndrome (gray line) in response (‐‐‐‐‐ line) and no response (‐ ‐ ‐ line) cohorts based on risk scores. (a) Paris‐1, (b) Paris‐2, (c) Barcelona, (d) Rotterdam, (e) Toronto, and (f) Mayo.

The 10‐year TFS for patients with PBC who had an optimal *versus* suboptimal treatment response was 92% (95% CI: 88.3–96.3) *versus* 74% (95% CI: 63.6–85.8) per Paris 1 (*P* < 0.0001), 95% (95% CI: 90.8–98.3) *versus* 79% (95% CI: 72.1–87.5) per Paris 2 (*P* = 0.0002), 93% (95% CI: 89.0–96.4) *versus* 65% (95% CI: 51.8–80.5) per Barcelona (*P* < 0.0001), 96% (95% CI: 92.4–98.7) *versus* 68% (95% CI: 57.5–79.8) per Rotterdam (*P* < 0.0001), 92% (95% CI: 87.8–95.9) *versus* 78% (95% CI: 68.3–88.2) per Toronto (*P* < 0.0008), and 91% (95% CI: 86.8–95.1) *versus* 77% (95% CI: 66.5–89.2) per Mayo (*P* = 0.003) risk scores (Fig. 4).

## Discussion

Since the development of PBC advanced risk models (GLOBE and UK‐PBC scores), little is known about the accuracy and reliability of their use outside Europe. In this analysis, we found that the Rotterdam criteria and both the UK‐PBC and GLOBE scores had good and comparable prognostic predictive values when used in PBC or AIH‐PBC overlap syndrome patients receiving UDCA. In addition, the UK‐PBC and GLOBE scoring systems were superior (C‐statistic of 0.7–0.75) to the other “treatment response” criteria (exclusive of the Rotterdam criteria) in predicting TFS of UDCA‐treated patients with PBC. Therefore, we conclude that the Rotterdam criteria, in addition to the UK‐PBC and GLOBE scores, are effective and can be generalized to the U.S. population.

In our study, we recruited 311 patients with a confirmed diagnosis of PBC only and 41 patients with AIH‐PBC overlap syndrome; the median overall age at diagnosis was 55 years, which is similar to the reported mean age at diagnosis in other western countries.^28^ However, on average, patients with adverse events were diagnosed approximately 3 years earlier than the noncomplicated PBC patients (52 *vs* 55 years). This finding is supported by a cross‐sectional study by Carbone *et al*.,^29^ where they analyzed biochemical data for 2353 patients with PBC receiving UDCA and found that the more symptomatic and less‐responsive disease to therapy was found in women presenting before the age of 50 years. The majority of patients involved in our study were females (85.8%),^30^ in line with the previously reported numbers in the western population. Among the studied patients, 13 (5.4%) developed PBC recurrence after liver transplantation in contrast to the conveyed PBC recurrence rate of 8–30%.^31^ This significant discrepancy could be related to the lack of uniform diagnostic criteria for PBC recurrence, the increased use of prophylactic UDCA after liver transplantation,^32^ and the advancement in posttransplant immunosuppressive therapy.^33^ Cirrhosis was present at diagnosis in 31.8% of our study patients compared to 28.5% of the involved cases in a study by Cheung *et al*..^34^ Expectedly, when stratified for baseline cirrhosis, the 10‐year OS was significantly lower among cirrhotic patients, 81 *versus* 94% (*P* = 0.0011). Notably, our cohort OS was slightly higher than that reported by the Lammers *et al*. meta‐analysis,^28^ with 92, 83, and 72 *versus* 90%, 78%, and 66% at 5, 10, and 15 years, respectively. This difference may be caused by early detection, improvement of PBC natural history, and advances in the management of liver‐related complications and liver transplantation in recent years.^35^ Although liver histology is not necessary for the diagnosis of PBC,^36^ it has an important prognostic significance.^37^ The studied cohort represents a heterogeneous sample of early histological disease (stages 0, 1, and 2; 63%) and advanced histological disease (stages 3 and 4; 41%). In comparison to the derivation group of the GLOBE study,^23^ where 39.5% had available liver biopsy results, among them, 55.9% had early disease, while 25.7% were found to have advanced disease. HCC was diagnosed in 0.28% of our involved patients, and this number is similar to the observed prevalence of 0.34% in a multicenter, international study by Trivedi *et al*.^38^ involving 4565 patients with a follow‐up period of more than 40 years.

In our study, evaluation of the advanced prognostic models demonstrated an overall performance (measured by C‐statistic) of 0.75 for the GLOBE score in contrast with 0.82 in the GLOBE validation cohort^23^ and an overall performance of UK‐PBC score of 0.70, 0.75, and 0.74 *versus* 0.96, 0.95, and 0.94 after 5, 10, and 15 years, respectively, in the prospective validation cohort.^22^ However, there was no statistically significant difference between the two scoring models as their CIs overlap. We also evaluated the performance of treatment response criteria after 1 year of UDCA treatment with the exception of the Toronto criteria, which was measured after 2 years of therapy, and the calculated C‐statistic ranged between 0.54 and 0.75. Among the above criteria, the Toronto criteria had the lowest overall predictive value, while the Rotterdam criteria outperformed the other criteria in predicting adverse events. The better performance of the GLOBE and UK‐PBC scores compared to the other treatment response criteria (with the exception of the Rotterdam criteria) might be due to the use of continuous, instead of categorical, variables, which include important factors that reflect liver synthetic function (albumin), portal hypertension/cirrhosis (platelet count), biliary disease (ALP and bilirubin), and the patient's age.^28^, ^29^, ^39^ Furthermore, the advanced models are based on baseline and 1‐year posttreatment disease analysis, while the treatment response criteria only evaluate the posttreatment disease.

Consistent with prior studies,^23^, ^34^ all biomarker prognostic models showed a relatively high negative predictive value (NPV) (>93% at 5 years, >87% at 10 years, and >84% at 15 years) but a low PPV (<19% at 5 years, <39% at 10 years, and <41% at 15 years) in predicting adverse events. Generally, as the disease progresses, the NPV decreases, while the PPV increases. Among treatment response criteria, the Rotterdam and Barcelona criteria are considered good predictors of good overall prognosis.

The present study has several limitations, including the retrospective nature of the data collection with its inherent biases. Moreover, our study represents a single tertiary center experience that is liver transplant‐capable, which might generate selection bias. However, manual review of all cases and application of strict inclusion and exclusion criteria largely contributed to eliminating questionable diagnoses to the greatest extent possible in a retrospective study. In addition, the long follow‐up period, with a median of 9.2 years, allowed for an accurate analysis of adverse events and liver‐related/overall mortality.

In conclusion, the Rotterdam criteria and the UK‐PBC and GLOBE scores outperformed the other risk scores for predicting OS and TFS in patients with PBC who were treated with UDCA for at least 1 year. In addition, they were both accurate and valid for use as prognostic models in the U.S. population.
